# The potential of microfluidic cell analysis in CAR T cell therapy manufacturing

**DOI:** 10.3389/fbioe.2025.1613836

**Published:** 2025-10-29

**Authors:** Aleksandra Nikoniuk, Koki Lilova, Michael Thomas, Nicolas Szita

**Affiliations:** ^1^ Department of Biochemical Engineering, UCL, London, United Kingdom; ^2^ Autolus Ltd., London, United Kingdom; ^3^ London Centre for Nanotechnology, UCL, London, United Kingdom; ^4^ Young Owl Ltd, London, United Kingdom

**Keywords:** cell therapy manufacturing, microfluidics, critical quality attributes, quality control, CAR T cell, process analytical technologies (PAT)

## Abstract

Despite significant success in treating hematological cancers, Chimeric Antigen Receptor (CAR) T cell therapies must overcome several challenges to become accessible to a wide patient population. With the high cost of treatment stemming partly from the complexity of the manufacturing process, there is a need for radical innovation in the ways those therapies are made. A crucial aspect of the manufacturing process is quality control (QC), responsible for monitoring the quality of the drug product. The use of microfluidic technology, in which microchannels are designed and fabricated to achieve high control of liquids, can increase sensitivity, lower the Limit of Detection (LoD), and improve time-to-result of analytical assays. This review examines how recently developed microfluidic devices for T cell analysis fit the requirements of QC testing in CAR T cell manufacturing. A particular focus is on cell counting, cell phenotyping, and cytotoxicity assessments, where a range of microfluidic approaches have been taken to deliver reliable analytical assays. The review not only highlights current limitations of microfluidic devices that hinder their implementation in manufacturing, but also their potential to expand on current QC testing.

## 1 Introduction

Chimeric Antigen Receptor (CAR) T cell therapies^1^ are revolutionizing the treatment of hematological cancers, achieving high remission rates in previously unresponsive patients (Dagar et al., 2023). As of March 2025, seven CAR T cell therapies have been approved by the U.S. Food and Drug Administration (FDA), and hundreds more are in clinical trials across the world. However, their prohibitive price of USD 500k restricts their use for the population at large. The complex and often labor-intensive manufacturing process itself costs USD 170-220k per batch, and therefore per patient (Khang et al., 2023). The process requires highly specialized cell manipulations, starting with T cell purification and activation, through viral transduction, expansion and, finally, cryopreservation. Currently, skilled labor is employed to carry out these steps under Good Manufacturing Practice (GMP). The lack of automation adds to the overall cost not only in terms of operator remuneration but also batch failures caused by human error (Lopes and Sinclair, 2020). The key to successful process execution is quality control (QC), which informs of critical quality attributes (CQAs) at relevant process stages as well as on the final product profile. The CQAs outlined in Table 1 can be divided into four categories. Safety encompasses attributes that can impact patient safety–absence of bacteria (sterility, *mycoplasma*) and genetic stability of the transduced cells (Vector copy number, VCN; Replication-competent retrovirus/lentivirus, RCR/RCL). Identity defines the product–what it looks like (visual appearance), dose (number of CAR T cells), and how many cells are viable (viability). Purity defines the cell content of the drug product–the attributes quantify the presence of wanted (T cells, CAR-T cells) and unwanted cells (e.g., B cells, leukemic cells) or reagents (magnetic beads). Potency describes the ability of the drug product to target cancer cells, which includes assessing the percentage of cells that are CAR^+^ (CAR^+^ expression), how well they kill target cells (*in vitro* cytotoxicity) and trigger an immune response upon target encounter (cytokine release). Although efforts have been made in the advances in automation of cell processing steps with the introduction of the CliniMACS Prodigy or Lonza’s Cocoon bioreactor systems, similar improvements have not yet been seen in QC (Mock et al., 2016; Trainor et al., 2023). As QC costs have been modelled to account for 32% of the cost of goods (COGs) (Harrison et al., 2019), a greater focus on analytical innovation could lead to significant savings.

**TABLE 1:** Critical Quality Attributes (CQAs) in CAR T cell manufacturing with example specification.

Category	CQA	Example specification	Reference
Safety	Sterility	Sterile	USP/Ph. Eur^a^
	*Mycoplasma*	Negative	USP/Ph. Eur^a^
	Endotoxin	<0.5-3.5 EU/mL	Machietto et al. (2023), Delgado et al. (2022)
	VCN	≤4 copies/cell	Hollyman et al. (2009)
	RCL	Negative	USP/Ph. Eur^a^
Identity	Visual appearance	Cloudy liquid	Delgado et al. (2022)
	Dose (from cell concentration)	>50 million CAR T cells	Rotte et al. (2022)
	Viability	≥70–80%	Hollyman et al. (2009), Delgado et al. (2022)
Purity	CAR^+^ expression	≥15%	Gee (2015)
	CD19^+^ cell content	≤2%	Gee (2015)
	CD3^+^ cell content	≥90%	Castella et al. (2020)
	Other impurities e.g. magnetic beads	<100 beads per 3 × 10^6^ cells	Hollyman et al. (2009)
Potency	CAR^+^ expression	≥15%	Gee (2015)
	Cytokine release	1–10 ng/mL	Castella et al. (2020)
	*In vitro* cytotoxicity	>20%	Gee (2015)

^a^ Testing requirements are outlined in the United States Pharmacopoeia (USP) and European Pharmacopoeia.

### 1.1 CQA assessments in CAR T cell therapy manufacturing

#### 1.1.1 Safety

In the early CAR T cell therapy clinical trials, safety testing was adopted from the pharmaceutical industry, following the compendial methods outlined in the United States and European Pharmacopoeias (Gebo and Lau, 2020). Sterility testing involves culturing samples in growth media for an extended period of time (14–28 days) and subsequently detecting contamination through either visual inspection or a colourimetric signal. BacT/Alert or Bactec are examples of FDA-validated, commercially available sterility testing methods that follow the compendial guidance, whilst standardizing the procedure (Lin-Gibson et al., 2021). However, the assay duration remains extensive, which contributes to the vein-to-vein time; hence, a recent focus has been on the development of rapid testing methods. Examples include the PCR-based detection of bacterial ribosomal DNA within 24 h (Van Den Bos et al., 2023) and the Microsart ATMP Sterile Release kit developed by Sartorius, which can deliver results in 3 h (Dias et al., 2024). Further approaches include mass spectrometry, Enzyme-linked Immunosorbent Assay (ELISA), next-generation sequencing (NGS), or flow cytometry-based methods, the advantages and disadvantages of which have been discussed elsewhere (Lin-Gibson et al., 2021).

The compendial *mycoplasma* detection method specifies three assays–culture in broth, agar, and in fluorescently labelled eukaryotic cells - and takes up to 28 days (Gebo and Lau, 2020). Rapid microbial methods based on nucleic acid amplification have been developed and are accepted by the regulatory authorities if appropriately validated (Marton et al., 2025). Those commercially available kits can deliver results in as fast as 1–5 h. To follow the strict GMP guidelines, the assays are carried out in specialized microbiology facilities, adding to the complexity and cost of manufacturing operations, as well as extending the time-to-result (Dias et al., 2024). Alternatively, the use of commercial kits can be implemented in-house; however, internal validation must be performed (Marton et al., 2025).

The compendial method for endotoxin detection utilizes the Limulus Amoebocyte Lysate (LAL), which, upon contact with endotoxin, forms a gel or produces a dye. The detection of this change indicates the presence of endotoxin in the sample. The assay has been incorporated into a cartridge compatible with detection via a hand-held spectrophotometer (Endosafe nexgen-PTS) for ease of operation (Barry et al., 2020). Validation of the assay for release testing is crucial, as the highly heterogeneous nature of the CAR T cell product can interfere with the LAL-endotoxin reaction (Marton et al., 2025).

#### 1.1.2 Potency

The Chromium-51 (^51^Cr) release assay is used to measure cytotoxic activity by detecting the radioactive chromium in the supernatant, following a co-culture of ^51^Cr-loaded target cells with effector cells (Cao et al., 2024). To improve operator safety and reduce radioactive waste, alternative detection methods such as flow cytometry or cell imaging are utilized following the target-effector (T-E) co-culture (Kiesgen et al., 2021). Flow cytometry offers endpoint analysis, where different cell populations are detected and compared to starting T-E ratios. Imaging systems like Incucyte offer real-time monitoring of cell death; however, they require fluorescent labelling of cells. The development of the xCELLigence platform, which utilizes impedance to detect cell killing in a co-culture, offers manufacturers label-free real-time data acquisition capabilities (Lisby et al., 2022).

Typically, cytokine secretion is detected using methods such as ELISA or ELISpot. Flow cytometry or the Luminex technology, which combines the two, can also be utilized (Marton et al., 2025). These are lengthy and labor-intensive assays; therefore, the development of automated immunoassays, such as ELLA developed by ProteinSimple, is a welcome improvement. The measurement of IFN-γ production following CAR T cell stimulation as an indicator of CAR T cell potency is accepted by the regulatory agencies; however, it is recommended that a cell killing assay and measurement of transduction efficiency be carried out as well (FDA, 2024).

#### 1.1.3 Identity and purity

Identity and purity assessments are performed utilizing a range of cell counting methods from Trypan blue (Hollyman et al., 2009) to automated cell counters (Liu et al., 2024). Flow cytometry is a key analytical technique to assess the expression of cell surface markers (Castella et al., 2020), which defines the purity of the cell population, e.g., how many of the cells are CD3^+^ and therefore T cells. The combination of cell counting and expression of surface proteins aids in calculations identifying the product, e.g., defining the CAR T cell dose.

### 1.2 Benefits of microfluidics

**FIGURE 1:**
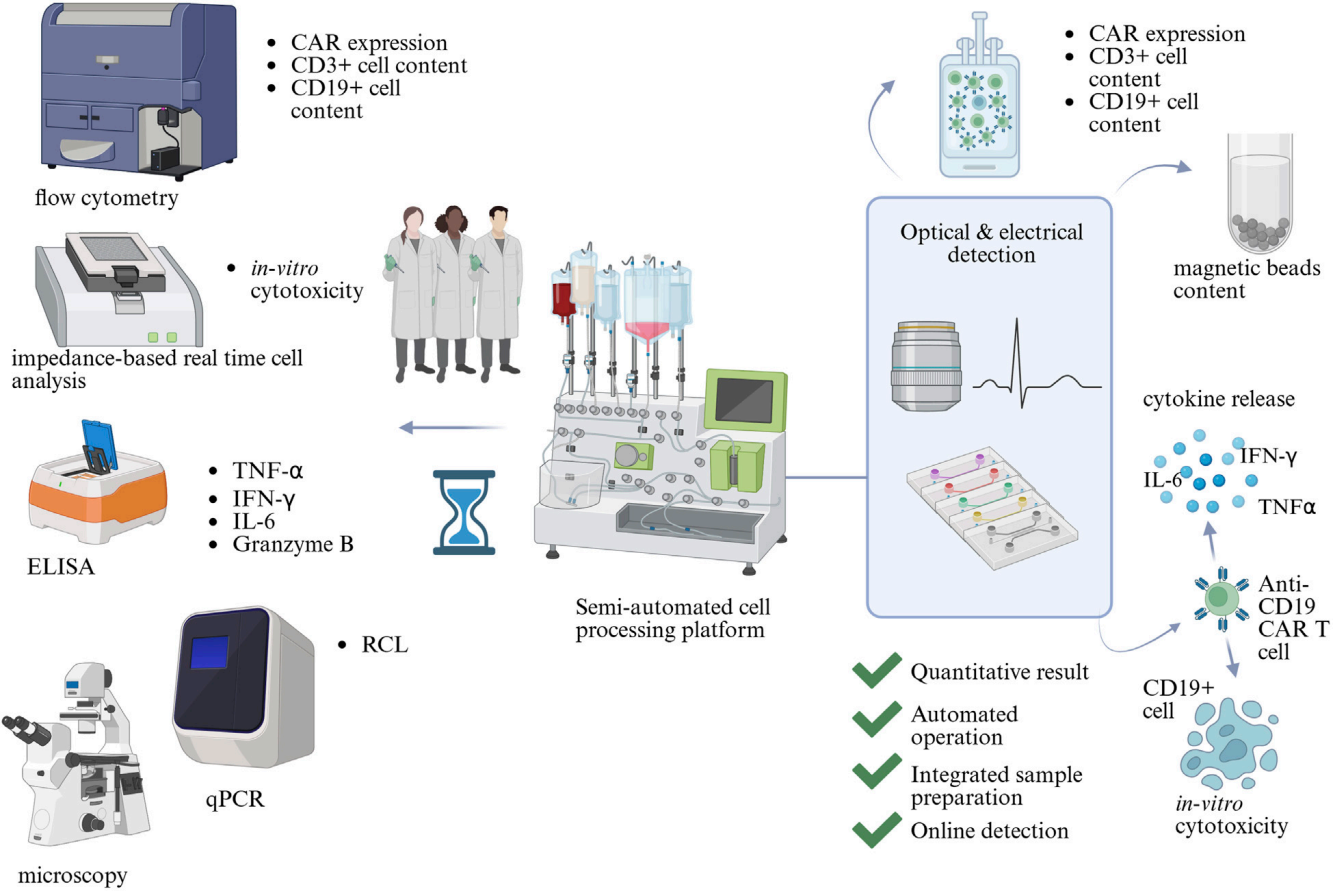
The potential of microfluidics in Critical Quality Attribute (CQA) assessment in CAR T cell therapy manufacturing. CQAs are assessed by a range of analytical methods, which include flow cytometry, microscopy, impedance-based real-time cell analysis, qPCR, and Enzyme-Linked Immunosorbent Assay (ELISA). They require trained scientists to operate and analyse results. Microfluidic technology could combine multiple analytical assays into automated devices with integrated readouts, decreasing the complexity of QC testing. Created in BioRender https://BioRender.com/wts7pai.

Microfluidic technology offers excellent spatiotemporal control, over the cellular microenvironment short diffusion path lengths, and operates at low volumes, meaning reduced use of resources (Sekhwama et al., 2024). These advantages can increase sensitivity, lower the Limit of Detection (LoD), and improve the time-to-result of analytical assays. Operating at the microscale could reduce sample and reagent use, which, when coupled with automation capabilities, could reduce costs associated with QC testing and ultimately CAR T cell therapies. The large number of analytical techniques could be combined in a microfluidic system as proposed in Figure 1. Unit operations such as Peripheral Blood Mononuclear Cells (PBMCs) separation from whole blood, T cell isolation, gene delivery, as well as functionality assessments, have already been translated into microfluidic devices (Aranda Hernandez et al., 2021; Wang and Kelley, 2025; Kim et al., 2024; Zia et al., 2024).

This mini review presents how different technologies, namely droplet microfluidics, surface cell capture, electrical detection, physical parameter detection, and microwells, are used in microfluidic devices to detect cellular attributes related to identity, purity and potency; safety testing is outside the scope of this review as its challenges are not T cell specific. Furthermore, the review assesses whether the devices, in their current format, can be readily integrated into CAR T cell QC testing routines or whether any shortcomings need to be addressed before successful implementation.

## 2 Droplet microfluidics as a tool for cell and reagent co-localization

Droplet microfluidics, a technique where sub-microliter droplets are generated, manipulated, and controlled within an immiscible carrier fluid, offers a range of applications for cell analysis (Li et al., 2023). Single cells or groups of cells can be enclosed within droplets, which allows for tracking of their behavior over time. Each droplet forms a distinct microscale cell culture chamber, offering a significant increase in experimental throughput.

### 2.1 Cytokine release quantification

Cytokine release is an indicator of CAR T cell functionality and is therefore one of the CQAs (Table 1). Cells and microbeads functionalized against IL-10 cytokine were co-encapsulated with a fluorescently tagged detection antibody to quantify cytokine secretion at concentrations as low as 10 pg mL^−1^ (Konry et al., 2011). Similarly, antibody-coated nanoparticles were enclosed inside a droplet alongside T cells and detection antibody, imaged, and analysed using an automated image processing algorithm (Bounab et al., 2020). Lu et al. (2024) utilized DNA proximity assay (DPA) within their microfluidic platform iSECRETE, capable of sample preparation, incubation, and quantification of IFN-γ at limits of detection (LoD) of 0.13 ng mL^−1^. Within 30 min, the signal was detected from processing 20 µL of a highly heterogeneous sample of whole blood. The system was expanded for CAR T cell cytotoxicity testing by incorporating further fluorescence markers within the droplet. These devices integrate sample processing and on-chip detection to provide sensitive results in the relevant range (Table 1), highlighting their potential as analytical systems for a manufacturing setting.

Surface-Enhanced Raman Spectroscopy (SERS) is a highly sensitive detection method that enhances Raman spectra when analytes of interest are in close proximity to plasmonically active metallic interfaces (Lian et al., 2025). It is used in the development of biosensors and offers the capability of both labelled and label-free detection formats. Its incorporation into microfluidic devices is understood to increase the reproducibility and accuracy of analyses because of the controlled conditions with which analytes are delivered and interface with plasmonic surfaces in the enclosed microfluidic environment.

In the context of cytokine detection, a relevant example of microfluidic labelled-SERS is the sandwich immunoassay developed by Cong et al. (2022) for the detection of vascular endothelial growth factor (VEGF). While for application in CAR T cell manufacturing, the VEGF antibody would have to be replaced with one against a relevant cytokine, e.g. IFN-γ, the assay for VEGF provided a highly sensitive result in the range of pg mL^−1^. Whilst sensitive, the approach required extensive cell sample preparation off-chip, two separate devices, as well as expensive and bulky confocal Raman detection equipment. When balancing assay sensitivity with its complexity and ease of operation, the latter might be the priority when introducing innovative solutions into CAR T cell manufacturing.

### 2.2 Cytotoxicity assessment

On top of cytokine secretion quantification, cell-based cytotoxicity assays are required by the regulators, which typically involve co-culture of CAR T cells with a cell line expressing the target surface marker. A droplet microfluidic device was developed to assess the killing potential of CD8^+^ T cells through measurement of calcium flux or machine learning-assisted detection of fluorescence (Sarkar et al., 2016; Sarkar et al., 2020; Sullivan et al., 2020). Despite providing a relevant analytical result, their device required sample preparation prior to the microfluidic assay as well as manual processing. Recently, a commercial microfluidic device was utilized to encapsulate CAR T cells with target cells for cytotoxicity testing, yet the assay readout was performed manually off-chip (Usheva et al., 2025). These devices show promise in delivering complex assessments of T cell killing potential, however, they fall short of integrating sample preparation, online detection, or assay automation (Table 2), critical in enhancing the efficiency of CAR T cell manufacturing.

**TABLE 2:** Microfluidic devices and their suitability to QC testing in the CAR T cell manufacturing setting.

System category	Critical quality attribute (CQA)	Assay	Quantitative result	Automated operation	Integrated sample preparation	On-chip detection	Reference
Droplet microfluidics	Potency	VEGF detection	Yes	No	No	Yes	Cong et al. (2022)
		T cell cytotoxicity	Yes	No	Yes	Yes	Ronteix et al. (2022)
		Detection of IFN-γ, T cell cytotoxicity	Yes	Yes	Yes	Yes	Lu et al. (2024)
		Detection of IL-10	Yes	Yes	Yes	Yes	Konry et al. (2011)
		Detection of TNF-α, IFN-γ, IL-2, IL-4	Yes	Yes	Yes	Yes	Bounab et al. (2020)
		T cell cytotoxicity	Yes	No	No	Yes	Sarkar et al. (2016), Sullivan et al. (2020), Sarkar et al. (2020)
		T cell cytotoxicity	Yes	Yes	No	Yes	Wong et al. (2022)
		CAR T cell cytotoxicity, detection of Granzyme B	Yes	Yes	No	No	Usheva et al. (2025)
	Purity	3D cell imaging	Yes	No	Yes	No	Cardenas-Benitez et al. (2024)
Surface modification for cell capture	Potency	Detection of IFN-γ and IL-2	Yes	Yes	Yes	Yes	Baganizi et al. (2015)
	Purity	CD4^+^ T cell counting	Yes	Yes	Yes	No	Cheng et al. (2007), Cheng et al. (2009)
		CD4^+^ T cell counting	Yes	No	Yes	Yes	Moon et al. (2011)
		CD4^+^ T cell counting	Yes	N/A	Yes	Yes	Kanakasabapathy et al. (2017)
		CD19^+^, CD4^+^ and CD71^+^ cell counting	Yes	Yes	Yes	Yes	Li et al. (2012), Liu et al. (2014), Zhang and Pappas (2016)
		CD4^+^/CD8^+^ cell counting	Yes	Yes	No	Yes	Li et al. (2015)
		CD64^+^. CD69^+^, CD25^+^ cell counting	Yes	Yes	No	Yes	Zhang et al. (2018), Zhou et al. (2019), Griffin et al. (2025)
		CD8^+^CD57^+^ cell counting	Yes	Yes	Yes	Yes	Choi et al. (2024)
Electrical detection	Potency	Jurkat dose-response cytotoxicity	Yes	No	No	No	Barbulovic-Nad et al. (2008)
		Detection of IFN-γ and TNF-α	Yes	N/A	Yes	Yes	Liu et al. (2011), Liu et al. (2015)
	Purity	CD4^+^ T cell counting	Yes	Yes	Yes	Yes	Phi et al. (2025)
		Lymphocytes, monocytes, and granulocytes counting	Yes	Yes	Yes	Yes	Liu et al. (2019), Liu et al. (2020), Civelekoglu et al. (2022a)
		CD4^+^ and CD8^+^ cell counting	Yes	Yes	Yes	Yes	Watkins et al. (2013)
		Detection of EpCAM	Yes	Yes	No	Yes	Civelekoglu et al. (2019), Civelekoglu et al. (2022c)
		Lymphocytes, monocytes, granulocytes counting	Yes	Yes	No	Yes	Civelekoglu et al. (2022b)
		CD4^+^ and CD8^+^ cell counting	Yes	Yes	Yes	Yes	Arifuzzman et al. (2023)
		Detection of apoptotic cells	Yes	Yes	Yes	Yes	Arifuzzman et al. (2025)
		CD4^+^ cell counting	Yes	Yes	No	Yes	Wang et al. (2008)
		Erythrocyte counting	Yes	Yes	N/A	Yes	Panwar and Roy (2019)
Physical properties detection	Purity	Monocyte and lymphocyte detection	No	Yes	N/A	No	Kang et al. (2018)
		CD45^+^, CD3^+^ and CD19^+^ cell detection	Yes	Yes	N/A	Yes	Noor et al. (2018), Mohd Noor et al. (2020)
		CD4^+^ and CD8^+^ cell detection	Yes	Yes	No	Yes	Rossi et al. (2019)
		Leukocyte counting	Yes	No	No	Yes	Hosokawa et al. (2012)
Microwells	Potency	Detection of TNF-α, IFN-γ, MCP-1, GM-CSF, IL-1β, IL-6	Yes	No	No	Yes	Ma et al. (2023)
		NK cell cytotoxicity	Yes	No	No	Yes	Park et al. (2019)
		Detection of Granzyme B, IFNγ, MIP-1α, TNF-α, GM-CSF, IL-2, IL-8, IL-4, IL-13, IL-22, IL-6, IL-17A	Yes	No	No	Yes	Xue et al. (2017)
		Detection of IFN-γ	Yes	N/A	Yes	Yes	Son et al. (2016)
		Cytotoxicity, detection of IFN-γ	Yes	No	No	Yes	Zhou et al. (2020)
		T cell cytotoxicity	Yes	N/A	No	Yes	Tu et al. (2020)
		Detection of IL-1α, IL-1β, IL-6, IL-8, IL-10, IL-12, IL-15, IL17A, TNF-α, IFN-γ, MCP-1	Yes	No	No	Yes	Song et al. (2021)
Other	Potency	T cell counting	Yes	No	No	Yes	Song et al. (2019)
		CD4^+^ T cell counting	Yes	Yes	Yes	Yes	Qiu et al. (2018)
	Purity	Capture of T cells for SERS	Yes	Yes	No	No	Dey et al. (2019)
		CD4^+^ T cell counting	Yes	Yes	No	Yes	Rodriguez et al. (2005)
		CD4^+^ T cell counting	Yes	No	No	Yes	Bae et al. (2009)
		CD4^+^ T cell counting	Yes	Yes	No	Yes	Frankowski et al. (2013)
		Macrophage cell line counting	Yes	Yes	No	Yes	Fan et al. (2019), Fan et al. (2020)
		Cell line counting	Yes	Yes	No	Yes	Liang et al. (2022)

### 2.3 Beyond CQA analysis

Some microfluidic systems are capable of quantification of relevant CQAs whilst also providing additional analysis. Wong et al. (2022) encapsulated CAR T cells with target cells for cytotoxicity measurement, and at the same time monitored CAR T response to target cells, such as whether the killing behavior was exhibited by single cells or clusters, or if CAR T cells expanded when exposed to their targets. However, image collection and analysis were manual, and the assay was tested using only two CAR T cell batches, indicating further validation is needed for employment in a manufacturing setting. Similarly, the device developed by Sarkar et al. (2020) is capable of not only cytotoxicity measurement but also monitoring of cell-to-cell contact times, the killing time, and the capacity for repeated killing. Microfluidic droplets have also been used to assess T cell killing behavior in complex tumor structures, which is relevant in developing analytics for CAR T cells targeting solid tumors (Ronteix et al., 2022). Cardenas-Benitez et al. (2024) developed a trapping device for 3D imaging of cells inside rotating droplets, providing detailed phenotyping data. However, to detect a specific T cell marker, the sample had to be fluorescently labelled off-chip. These examples show that microfluidic technology can assess relevant CQAs while providing additional information; however, it remains to be seen whether such additional testing will ever become a requirement in CAR T cell process monitoring and release testing.

## 3 Microfluidic surface modification for cell capture and detection

One approach to microfluidic cell quantification is target cell capture via antibodies immobilized on a device’s surface, followed by detection of either bound or unbound cell fraction. This approach was taken to quantify CD4^+^ T cells by counting surface-bound cells using microscopy (Cheng et al., 2007; Cheng et al., 2009). Alternatively, Charge-Coupled Device (CCD) cameras (Moon et al., 2011) or smartphones (Kanakasabapathy et al., 2017) can also be implemented to image surface-bound CD4^+^ T cells, with software integrated for image processing. Good statistical agreement was shown between the microfluidic methods and flow cytometry, as well as good sensitivity (over 80%) at relevant sample concentrations (up to 5 × 10^6^ cells mL^−1^). Such fast (10–30 min), low-volume (10–50 µL), low-cost, and simple to operate devices for immune cell enumeration are exactly what is needed in a CAR T cell manufacturing setting to decrease QC costs. For effective CQA monitoring, detection of different cell types is required, ideally all within one microfluidic device.

The Pappas Group has developed microfluidic devices that capture target cells on surfaces functionalized with antibodies against cell surface proteins (Li et al., 2012). They utilize polydimethylsiloxane (PDMS) stamping or pneumatic valves to coat specific areas of microfluidic channels with biotinylated antibodies, forming discrete cell-capture regions on the surface (Li et al., 2012; Liu et al., 2014). This allows detection of T cells expressing different cell surface markers, cells expressing different levels of the same antigen (Zhang and Pappas, 2016) as well as CD4^+^/CD8^+^ ratio determination (Li et al., 2012; Li et al., 2015) and counting of CD64^+^ and CD69^+^ cells in patient samples to determine their sepsis status (Zhang et al., 2018; Zhou et al., 2019; Griffin et al., 2025).

Cytokine secretion analysis was performed on antibody-coated surfaces, where antibodies were used for both cell immobilization and cytokine capture. The signal from bound cytokines at levels below 1 ng mL^−1^ was detected via Surface Plasmon Resonance Imaging (SPRI) and fluorescence microscopy (Baganizi et al., 2015). A combination of surface capture and fluorescent labelling can also be used to address the challenge of phenotyping specific cell types based on the expression of more than one marker. CD8^+^ T cells were captured on an antibody-functionalized PDMS-on-glass chip, followed by the flow of CD57 antibody on the captured cells to detect the proportion of CD8^+^ T cells that also express CD57 (Choi et al., 2024). A 10 µL heterogeneous sample of whole blood was tested, and the results were compared to flow cytometry, with good correlation, highlighting the efficiency and accuracy of this microfluidic system.

Surface capture of target cells coupled with optical detection and subsequent quantification is a promising approach for cell phenotyping. This could have a transformative impact on the quality control in CAR T cell manufacturing, where T cell markers such as CD3, CD19 or CAR define the product’s purity and potency. The key to implementing microfluidic phenotyping systems in a manufacturing setting lies in workflow automation and the capability to detect multiple cell markers within one device or several devices operated simultaneously.

## 4 Cell phenotyping and cytokine detection using electrical signal

A Coulter Counter is an analytical technique used for counting blood cells by detecting changes in impedance of cell suspension flowing through an electric field (Grimnes and Martinsen, 2008). Watkins et al. (2013) captured CD4^+^ and CD8^+^ leukocytes from a whole blood sample and quantified them through the difference in impedance measurements pre- and post-capture chamber. The results were in agreement with flow cytometry, within a wide cell concentration range (0.04−1.3 × 10^6^ cells mL^−1^). Recently, Phi et al. (2025) utilized impedance measurements and microfluidic channels to develop a handheld device for CD4^+^ T cell enumeration of samples within the 0.075−1.2 × 10^6^ cells mL^−1^ range. Such devices show the potential of microfluidic systems for accurate and automated cell phenotyping.

Impedance measurements have been utilized alongside other microfluidic manipulations. The Sarioglu group combined surface modification for selective cell capture alongside electrical detection of uncaptured cells to determine proportions of different leukocytes in a sample (Liu et al., 2019; Liu et al., 2020; Civelekoglu et al., 2022a). Fluorescent tagging has been used to perform absolute counts of CD4^+^ T cells from a lymphocyte sample (Wang et al., 2008). Magnetic labelling has been utilized to detect cells expressing different amounts of CD33 protein on their surface (Civelekoglu et al., 2022b). Leukocytes were magnetically separated on-chip and enumerated with an integrated Coulter Counter. The device was further developed to introduce a feedback loop for operational automation (Civelekoglu et al., 2022c), highlighting the robustness of the system. Nonetheless, this approach might not be suitable for testing more homogeneous samples such as pure T cells. Additionally, to truly improve the operational efficiency of CAR T cell analytical testing, the labelling of samples must be done on-chip, which was not the case in this system.

A label-free Coulter counter for immune cells was further developed by Arifuzzman et al. (2023) and Arifuzzman et al. (2025), who utilized surface modification for cell capture to enumerate CD4^+^, CD8^+^ and apoptotic T cells. The assay operated with a low error of 7% and delivered results in close correlation to flow cytometry. Combining a Coulter counter, a label-free cell determination, and a feedback loop control system resulted in a microfluidic device that has a clear potential in a manufacturing setting, as it delivers a process-relevant result in an automated manner.

Gold electrodes were functionalized with cytokine-specific aptamers (IFNγ, TNFα) for the detection of the said cytokines by tracking changes in redox peak upon aptamer-target binding (Liu et al., 2011; Liu et al., 2015). A limit of detection (LoD) of 5.45 ng mL^−1^ was achieved, and due to the use of aptamers instead of antibodies, the system did not require washing steps. Electrodes were employed to actuate microfluidic droplets to assess the dose response of Jurkat cells to a toxic component. The device showed a 20-fold increase in assay sensitivity, with no negative impact of electrical manipulation on cell viability (Barbulovic-Nad et al., 2008).

Despite offering accurate and often label-free cell analysis, these devices required the integration of electrodes, making the fabrication more complex and expensive. As CAR T cell therapy manufacturing is already expensive, new QC testing methods are only viable if they reduce costs. It is therefore encouraging that simpler and more cost-effective solutions are being developed by utilizing cheaper fabrication methods and detection systems (Phi et al., 2025; Panwar and Roy, 2019).

## 5 Cellular analysis via microfluidic detection of physical properties

Label-free analytical techniques simplify the assay by removing the need for sample manipulation. Microfluidic technology has been used to distinguish between cell types based on their physical characteristics. Using stiffness, for example, a microfluidic pillar array with progressively decreasing distances between pillars forced cells to deform increasingly as they travel through (Kang et al., 2018). The device enabled discrimination between T cells and monocytes as well as CD4^+^ and CD8^+^ T cells. Although the capability to separate monocytes as well as CD4^+^ and CD8^+^ lymphocytes is impressive, there was no on-chip detection of sorted cells, which (currently) limits the analytical power of this approach.

Counting of CD4^+^ and CD8^+^ cells was carried out by passing a fluorescently stained whole blood sample through microcavities, where leukocytes were trapped based on their size, and other cell types were washed away (Hosokawa et al., 2012). Similarly, decreasing pore sizes inside a microfluidic device were used to trap CD45^+^ cells for image-based enumeration, which showed good agreement with flow cytometry results (Noor et al., 2018; Mohd Noor et al., 2020).

Alternatively, light scattering was used to collect physical information about cells, such as cell dimensions, refractive index of the nucleus and the cytosol, and the nucleus to cytosol ratio (Rossi et al., 2019). To enhance the differences and therefore improve detection capabilities, a stimulus was added to the sample before the information was fed into a machine learning model to distinguish between CD4^+^ and CD8^+^ cells. It is an example of how microfluidic sample processing can be integrated with artificial intelligence (AI) to deliver new methods of cell phenotyping. Despite some success in microfluidic cell type determination of relevant cell types (CD4^+^, CD8^+^, CD45^+^) using physical parameters, it is unclear whether enough physical differences can be detected between highly similar cells, e.g., CD3^+^CAR^+^ and CD3^+^CAR^−^ cells. The detection of physical parameters might therefore not be the most suitable microfluidic technology for integration into CAR T cell testing devices.

## 6 Use of microwells for cells and reagent co-localization

The use of microwells in microfluidic devices is widespread, enabling cell or reagent entrapment in a defined position for manipulation or analysis. A device with a collapsible roof containing microchambers was used to trap stimulated CD4^+^ T cells with beads functionalized with cytokine-specific and detection antibodies, all within one chamber (Son et al., 2016). The intensity of the fluorescence signal as the bead binds the IFN-γ cytokine was quantified, generating a single cell secretion profile. Xue et al. (2017) developed a single-cell barcode chip (SCBC) where stimulated CAR T cells were trapped inside wells exposed to a 16-plex antibody array for a range of cytokines involved in effector, stimulatory, regulatory, and inflammatory functions. Fluorescence signal was generated using detection antibodies and data analyzed using Isoplexis software.

Zhou et al. (2020) used microwells to assess T cell cytotoxicity against a prostate cancer cell line, while concurrently monitoring IFN-γ release through trapping of antibody-functionalized beads with the cell pair complex. Similarly, microwells were used to co-localize T cells with target leukemia cells for microscopy monitoring to investigate not only cytotoxicity but also the impact of contact time and distance on the killing potential, and the cell-to-effector ratios (Tu et al., 2020).

Whilst hematological cancers are present in solution (i.e., blood), solid tumors form complex 3D tumor microenvironments (TME), hampering the effectiveness of CAR T cells *in vivo*. Park et al. (2019) utilized a microwell-based microfluidic device to carry out a CD8^+^ T cell cytotoxicity assay on cells embedded in extracellular matrix (ECM). When compared to a standard 2D culture-based assay, the cytotoxicity was lower in the former, which could indicate that the 3D model is more representative of the TME. Ma et al. (2023) measured the CAR T cell release of six cytokines in media using plasmonic scattering signal from immobilized gold nanoparticles (AuNPs). The assay was carried out in a Leukemia-on-a-Chip microfluidic model, providing further complexity to the analysis. To successfully translate CAR T cell therapies against solid tumors to the clinic, analytical assays that are representative of target TME are needed. This is where microfluidic technology could prove crucial. Additionally, assays that deliver results for more than one CQA (e.g., cytotoxicity and cytokine release), or provide additional information not currently investigated as part of the standard routine, could become part of testing protocols in the future. However, despite delivering sensitive results in T cell potency testing, microfluidic microwell devices often lack integrated automated sample processing and online detection (Table 2). This limits their potential for implementation in a manufacturing setting, as operator intervention is still required. Integration of automated liquid handlers (Tran et al., 2022) or bespoke robotic systems (Li et al., 2014) could improve the prospects of microwell device implementation in a production environment.

## 7 Other

Alternative approaches have been taken to deliver results relevant to CAR T cell therapy manufacturing. One example is the cost-effective and straightforward CD4^+^ T cell counting, utilizing fluorescent antibody labelling alongside image cytometry (Rodriguez et al., 2005; Bae et al., 2009). Similarly, cell enumeration was obtained inside a microfluidic device by flowing magnetically pre-labelled cells into detection (target) and waste (non-target) zones (Song et al., 2019). Target cells were subsequently detected using a resistive pulse sensor. An alternating current electrohydrodynamic (ac-EHD) microfluidic device was used for the isolation of CD4^+^ T cells, where 100 target cells were detected within a heterogeneous sample of 106 cells. In addition, T cell receptor profiling was carried out using SERS (Dey et al., 2019). Despite the labelling of the sample with SERS tags, the SERS analysis was performed off-chip, thus without integration of sample processing with online detection.

While delivering accurate cell enumeration methods, these systems require sample pre-processing. This limits their applicability in manufacturing as any manual processing adds operational complexity.

A more suitable approach was developed by Qiu et al. (2018) where a single magnetic bead functionalized against CD4 was used for microfluidic CD4^+^ T cell quantification. Each step of a chemiluminescence sandwich immunoassay was integrated into the device, with magnetic control used not only to actuate the bead from one step to another but also to improve reagent mixing. As limited sample processing is required, this device is an excellent candidate for implementation in QC testing, provided that additional T cell markers can be quantified in this manner.

Flow cytometry is based on the flow focusing of cells into a single stream for the detection of surface proteins. This process can be miniaturized utilizing microfluidic technology and has been reviewed elsewhere (Gong et al., 2019). Despite advantages over flow cytometry in terms of reagent use and portability, microfluidic versions continue to rely on lasers and other high-cost detection equipment, which may reduce their practicality for implementation in already costly cell therapy manufacturing.

## 8 Summary

A range of microfluidic techniques, combined with various detection modes, has proven successful in establishing T cell analytical assays relevant to CAR T cell manufacturing. Some of the devices discussed here offer quantitative results with on-chip detection, coupled with automation and integrated sample preparation, making them attractive candidates for implementation in QC testing. In addition to comparable assay performance with the gold standard, further considerations will need to be addressed for microfluidics to have a significant impact on QC in CAR T cell manufacturing. These include, for example, specific requirements of the assay type, such as the stringent sterility demands in cytotoxicity testing, as well as microfluidics considerations, including the robustness of operation. Ultimately, the cost of device manufacture is also a factor, yet is outside the scope of this review. Furthermore, some devices demonstrate the capability to measure, for example, a specific cell marker, while others provide analytical readouts of multiple CQAs, such as cytotoxicity and cytokine secretion profiles. This raises the possibility of developing a device that incorporates all essential QC tests. However, integrating all tests into one device would pose a significant demand on the complexity of operation. It remains to be seen whether such systems can be straightforward to use and meet the demands of reproducibility required in the GMP environment. Comparing emerging technologies, such as microfluidic approaches, systematically with established methods used in industry would further support the development of such novel approaches; however for such a comparison, more data is needed about the existing assays. Improved transparency for example through peer-reviewed assessment of sensitivity, accuracy and cost-effectiveness of existing assays could provide such data. Additionally, direct interfacing with the cell processing systems for at-line or online CQA detection is the goal of developing Process Analytical Technologies (PATs). While microfluidic technology is available to deliver relevant analytical results, none of the devices discussed in this review have been reported to connect to processing equipment. The integration of automated microfluidic devices with the bioreactor systems will be crucial to truly realize the vision of PAT and drive the technological innovation in CAR T cell manufacturing.

## Footnotes

1 See glossary for full list of abbreviations.

## Glossary

ATMP: Advanced Therapy Medicinal Products: Medicines based on genes (gene therapy), cells (cell therapy), or tissues (tissue-engineered therapy) to repair or replace damaged functions
CAR: Chimeric Antigen Receptor: a receptor which is engineered into a T Cell in order to recognize a specific antigen on cancer cells
CCD: Charged-Coupled Device: a semiconductor chip which translates light into electrical charge, for example, in a CCD camera
CD3: Cluster of Differentiation; name and classification system for cell surface molecules, i.e. markers, for example, on white blood cells
COGs: Cost of Goods: the direct costs necessary to produce goods; for ATMPs, COGs include raw materials (e.g. viral vectors), labor (e.g. highly trained technicians), manufacturing facilities (GMP clean rooms), patient-specific costs such as cell collection and cold chain storage
CQA: Critical Quality Attributes: a property of a product that must be controlled to ensure safety, efficacy, and quality of the product; see Table 1 for CQAs in CAR T cell manufacturing
DPA: DNA proximity assay: an assay to detect whether molecules are close to each other; converts proximity into a DNA signal
ECM: Extracellular Matrix: the three-dimensional network of proteins and polysaccharides surrounding the cells, providing structural and biochemical support
EHD: Electro-Hydrodynamic: a principle enabling fluid motion by using an electric field, such as pumping liquid in a microfluidic channel
FDA: Food and Drug Administration: US government agency responsible for approval of medicines
ELISA: Enzyme-Linked Immunosorbent Assays: an antibody-based test which detects and quantifies biomolecules by using an enzyme-linked color change
GM-CSF: Granulocyte-Macrophage Colony-Stimulating Factor: a cytokine which promotes the growth of granulocytes and macrophages
GMP: Good Manufacturing Practice: a set of regulations ensuring medicines are consistently produced and controlled to quality standards
Granzyme B: A cytotoxic protease released by T cells and NK cells to induce target cell death
IFN: Interferon: cytokines that boost antiviral defence and regulate immune responses
IL: Interleukin: family of cytokines that mediate communication between immune cells
LoD: Limit of Detection: the lowest concentration of a substance that can be reliably detected by the assay
MIP: Macrophage Inflammatory Protein: Chemokines that recruit immune cells to sites of infection or inflammation
NGS: Next Generation Sequencing: High-throughput DNA/RNA sequencing technology for comprehensive genetic analysis
NP: Nanoparticle: Nano-sized particle used in drug delivery, imaging, or diagnostics; made of different materials, for example gold nanoparticles (AuNP)
PAT: Process Analytical Technology: A framework for designing, analyzing, and controlling biomanufacturing processes through real-time measurement of critical quality attributes and performance parameters
PBMC: Peripheral Blood Mononuclear Cells: A heterogeneous population of blood cells with round nuclei, including lymphocytes (T cells, B cells, NK cells) and monocytes
PDMS: Poly(dimethylsiloxane): a silicone-based polymer widely used in microfluidics and biomedical devices
QC: Quality Control: Procedures to ensure products meet defined safety and performance standards
RCL: Replication-Competent Lentivirus: Lentivirus capable of replicating, monitored as a safety risk in gene therapy
RCR: Replication-Competent Retrovirus: Retrovirus capable of replication, checked as a safety risk in retroviral vector use
SERS: Surface Enhanced Raman System: A sensitive spectroscopic method that enhances Raman signals using nanostructured surfaces
SPRi: Surface Plasmon Resonance Imaging: real-time, label-free optical analysis method for analyzing interactions between molecules
T Cell: T cells, also known as T lymphocytes, are a type of white blood cell that develops in the thymus gland (hence the T for thymus
T-E: Target-Effector: ratio describing target cells (e.g., tumor cells) versus effector cells (e.g., T or NK cells) in cytotoxicity assays
TME: Tumor Microenvironment: the cellular and molecular environment around a tumor that influences its growth and immune evasion
TNF: Tumor Necrosis Factor: a pro-inflammatory cytokine involved in immune defence and inflammation
VCN: Vector Copy Number: The number of therapeutic vector DNA copies integrated into the genome per cell
VEGF: Vascular Endothelial Growth Factor: A signalling protein that promotes new blood vessel formation
